# Dynamic educational recommender system based on Improved LSTM neural network

**DOI:** 10.1038/s41598-024-54729-y

**Published:** 2024-02-22

**Authors:** Hadis Ahmadian Yazdi, Seyyed Javad Seyyed Mahdavi, Hooman Ahmadian Yazdi

**Affiliations:** 1 Department of Computer Engineering, Neyshabur Branch, Islamic Azad University, Neyshabur, Iran; 2 Department of Electrical Engineering, Mashhad Branch, Islamic Azad University, Mashhad, Iran; 3 Department of Computer Engineering, Ferdowsi University of Mashhad, Mashhad, Iran

**Keywords:** Deep learning networks, Recurrent methods, Educational resource recommender system, Engineering, Electrical and electronic engineering

## Abstract

Nowadays, virtual learning environments have become widespread to avoid time and space constraints and share high-quality learning resources. As a result of human–computer interaction, student behaviors are recorded instantly. This work aims to design an educational recommendation system according to the individual's interests in educational resources. This system is evaluated based on clicking or downloading the source with the help of the user so that the appropriate resources can be suggested to users. In online tutorials, in addition to the problem of choosing the right source, we face the challenge of being aware of diversity in users' preferences and tastes, especially their short-term interests in the near future, at the beginning of a session. We assume that the user's interests consist of two parts: (1) the user's long-term interests, which include the user's constant interests based on the history of the user's dynamic activities, and (2) the user's short-term interests, which indicate the user's current interests. Due to the use of Bilstm networks and their gradual learning feature, the proposed model supports learners' behavioral changes. An average accuracy of 0.9978 and a Loss of 0.0051 offer more appropriate recommendations than similar works.

## Introduction

In recent years, educators' use of learning resources has increased significantly. Many educational resources are distributed in different repositories that respond to a wide range of various educational topics and goals. However, due to information overload, many learners have difficulty finding relevant and valuable learning resources that meet their learning needs^1^.

Although existing recommender systems have been very successful in the field of e-commerce, there are challenges, such as differences in learners' characteristics such as learning style, knowledge level, and learning pattern in creating an accurate recommender of learning resources in the field of e-learning^2^. Many existing recommendation methods do not consider differences in the learners' characteristics. Of course, this problem can be improved by adding plugin information about learners to the recommendation process. In addition, many recommendation methods face issues such as cold start and data sparsit^2^

In recent years, deep learning has gained significant results in different problems^3,4^.

Recently, with the help of deep learning methods, recommender systems have grown significantly. Recurrent neural networks (RNNs) play an essential role in modeling session-based recommender systems and promise further improvements. Compared to many other recommender models, an RNN uses a sorted sequence, or a consistent history of users, to create a comprehensive user profile that helps improve the recommendation^5–7^.

So far, much work has been done in combining long-term and short-term user interests^8–10^. On the other hand, user preferences for items evolve continuously over time^11^, while these networks implicitly assume that user interests are static, which is a false assumption in many scenarios^5,6,8,12^. Therefore, these methods work very poorly when the interests are content-sensitive and transient. There are also session-based methods^6,12^ that deal with interest drift to solve the problem of the static interests of the user. In contrast, these methods assume that all items have the same effect in a session, which is a false assumption, especially considering user interactions with items with different properties. We propose a long-term and short-term attention-based model to recommend educational resources to solve this problem. In none of the mentioned studies, an educational recommender that automatically provides useful advice has been developed; however, such a recommender is very much needed in educational applications. Our model recommends better training resources using a hybrid attention-based RNN network that simultaneously includes two types of data (short-term and long-term users' interests and updating educational resources). We considered the attention-based technique on the short-term interests of the user, and in the compression phase, we weighed the user interactions based on the time vector. That is, the more practical attributes of the user were considered to have a more effective effect on the recommendation of the educational resource. Unlike clustering techniques that ignore the fewest iterations, all user interactions are preserved. In fact, earlier works used only the user's previous information for education. While having a network that looks both backward and forward in this study allows us to cover changes in the learner's behavior and suggest updated recommendations. Utilizing a bidirectional LSTM deep neural network involves the user's long-term and short-term interests. We expect the proposed method to improve the recommendation of the next item, especially at the beginning of sessions, and to deal with the problem of a cold start at the beginning of each session.

The study of session-based recommender systems is not a new research topic^7,8,13^ and is more suitable for learning dynamic and sequential user behaviors compared to traditional iterative systems. The recurrent systems aim to generate search results close to the user's needs and make predictions based on their priorities. In virtual learning environments, educational recommender systems carry learning objects based on the student's characteristics, priorities, and learning needs. A learning object is a unit of educational content that, once found and retrieved, can assist students in their learning process^14^. According to the IEEE^3^ definition, a learning object is a digital or non-digital entity with educational design features that can be used, reused, or referenced during computer-based training.

Because of the ability of deep learning to solve many tasks and produce excellent results, universities and industries compete with each other to apply deep learning in a wider range of applications^15^. Recently, deep architectural learning has dramatically transformed recommenders and improved their performance. Deep learning can effectively capture non-linear and non-trivial user-item relationships and encode more complex abstractions as high-level data representations. In addition, it can obtain complex relationships within data from other sources, such as conceptual, textual, and visual information^15^. The increasing volume of data in the big data age poses challenges for real-world applications. As a result, scalability is essential for the efficiency of recommendation models in real-world systems, and temporal complexity significantly affects model selection. Fortunately, deep learning is very effective and reliable^16^.

## Literature review

In the following, the studies performed on educational recommenders will be reviewed.

### Data mining methods

Many educational researchers focus on extracting information about learning progress to help students properly. Yago et al.^15^ introduced an ontology network-based student model for multiple learning environments (ON-SMMILE), which is a semantic web-based model to assist teachers in educating students^13^. It is a constructive learning model in which students are highly involved in learning. This student model provides sufficient assistance to obtain, analyze, and categorize meaningful information about students and their knowledge status. It can also identify possible weaknesses and mistakes in learning. This helps the educator decide what recommendation should be given to each student. One of the advantages of this model is the possibility of applying different processing methods. Applying a combination of supervision and data processing methods to education provides a consistent and broad automated view of student learning.

Knowing the information that helps us define the user profile and identify their interests is essential to producing a personal recommender. Qiao and Xu^7^ introduced an infrastructure that can extract user-profiles and educational content from the Facebook social network and suggest educational resources to him/his^17^. This is done by information extraction and semantic web methods to extract, enrich, and define the user profile and interests. The recommendation action is based on repositories of learning objects, related data, and video repositories and takes advantage of how long the user has been using the Web. Evaluating the user's profile and behavioral characteristics via social media has the advantage that relevant educational information can be offered to him/her that will lead to his/her academic success. In addition, through this, the user's personal information and even his/her educational interests are always up to date. Of course, there are challenges, such as access to information and information extraction methods.

Tarus et al.^2^ introduced a hybrid knowledge-based recommender system based on ontology and sequential pattern mining to recommend e-learning resources to learners^2^. An ontology is used to represent knowledge about learners and educational resources, while the SPM algorithm is used to discover the sequential learning patterns of learners. One of the advantages of this method is that it can reduce the problem of cold start and sparsity.

Kuznetsov et al.^40^ showed how the ontology of students' skills could reduce the problem of the cold start of educational recommender systems. Students' profiles are created using their results in university courses^18^. The aim is to offer students projects and industrial opportunities. In this work, they used collaborative filtering algorithms and rule-based recommendations derived from user interactions in their system. The profile-based recommendation has been used to overcome the problem of cold start.

Since the learners' profiles are different, to use recommender systems in issues such as personal learning in the field of e-learning, an educational program should be prepared according to the needs of the learners. With knowledge of learners' profiles, more appropriate learning objects can be recommended to improve the learning process. Bourkoukou and Bachari^17^ proposed a LearnFit system that can automatically adapt to learners' dynamic priorities^19^. This system recognizes different patterns of learners' learning methods and habits by testing their psychological model and extracting their web browsing reports. In this system, to overcome the problem of cold start, which is consistent with the learning method of the learner, a personalized learning scenario is first obtained using the Felder-Silverman model^20^. Then, the habits and priorities of the users are obtained using data mining methods. Finally, the learning scenario is reviewed and updated by combining the collaborative filtering method and association rule mining.

Ludewig and Jannach^11^ introduced a recommender system that helps learners in choosing their lessons^21^. Choosing the right lessons in the early years can improve the research process. In this combined method, the N-gram query classification and the ontology are used to retrieve useful information and make an accurate recommendation. Generally, the query is converted to N-gram in the preprocessing step. The query extension is then applied using WordNet to retrieve similar words. They are then retrieved, and duplicate lessons are removed. In the last step, the related lessons are extracted using the ontology of the lessons. This method is faster than classical methods and helps learners to improve their performance and level of satisfaction and have easier access to information.

Recommendation systems (RSs) have been used and adapted in education as a means of offering each learner the educational resources best suited to his or her profile or needs, as is done in the field of e-commerce According to the filtering methods, the RSs are based upon two essential techniques: the first focused on the specificities of the user and the second focused on the preferences and tendencies of the group to which the user belongs. In order to improve the quality of recommendations in education, the author’s article^1^ has experimented with a hybrid approach that combines content-based and the aborative filtering approaches. This article uses an experiment to determine whether hybridizing content-based and collaborative filtering methods can improve the relevance of recommendations in an online educational context. The results demonstrated that the hybrid recommendation approach works best when considering the public institute alone and when considering the public and private institutes together. It can be concluded that taking into account both the individual and social specificities of a learner, can improve the relevance of the recommendations of educational resources in an e-learning environment.

In order to ensure the quality of resource recommendation and solve the problems of low recommendation accuracy, long recommendation time, and high data loss rate in the process of resource recommendation in traditional methods, a personalized recommendation system of English teaching resources based on the multi-K nearest neighbor regression algorithm is designed.

According to the overall architecture of the personalized recommendation system of teaching resources, in^2^ designs the resource browsing function module, teaching resource detailed page recommendation module, and teaching resource database. Based on the basic idea of the multi-K nearest neighbor regression algorithm, in order to avoid the loss of important data in English teaching resource recommendation and reduce the data loss rate, a missing data reconstruction algorithm for English teaching resources is proposed. Finally, the path interest of student users is considered from the selection of browsing path and access time to realize the personalized recommendation of English teaching resources. experimental results show that the system has high resource recommendation accuracy, short recommendation time, and low data loss rate in^13^ focuses on the collaborative filtering algorithm and proposes a collaborative filtering recommendation algorithm with an improved user model.

Firstly, the algorithm considers the score difference caused by different user scoring habits when expressing preferences and adopts the decoupling normalization method to normalize the user scoring data; secondly, considering the forgetting shift of user interest with time, the forgetting function is used to simulate the forgetting law of score, and the weight of time forgetting is introduced into user score to improve the accuracy of recommendation; finally, the similarity calculation is improved when calculating the nearest neighbor set. Based on the Pearson similarity calculation, the effective weight factor is introduced to obtain a more accurate and reliable nearest neighbor set.

experimental results show that the proposed method has better performance in recommendation accuracy and recommendation efficiency.

### Development of traditional methods

Traditional online learning systems are based on different filtering methods that often rely on user behavior in the face of different sources. Recommending resources extracted from users with similar behavior often does not have satisfactory results.

Hagemann et al.^20^ proposed a personal recommender to help students make informed decisions about their learning path^22^. This study aimed to improve the path of discovery of selected modules by students using a hybrid recommender system explicitly designed to help students better discover available options. By combining content-based similarity and dispersion based on structural information about module space, it is possible to improve the predictability of choices that are exclusively consistent with students' priorities and goals. One of the advantages of this is that it can add diversity to the set of recommendations.

Tseng et al.^21^ introduced an adaptive learning and recommendation platform as a tracking tool for educators to observe and monitor student learning activities^23^ Students can learn ALR using the learning path through the platform. The strengths and weaknesses of students' learning can be revealed through the analysis of their learning activities, learning process, and learning efficiency. The aim is to create a concept map for adaptive learning to provide an educational advisor for students. Alinani et al. (2016) proposed a heterogeneous educational resource recommender system based on user preferences^24^. This system not only meets users' needs but also reduces some of the problems of most recommender systems, such as cold start. To do this, the system recommends the user on the recent request process and learns from the user's behavior in the process. A key point of this is that each recommended resource is assigned a weight, which is calculated based on the user's response. Heterogeneous recommender resource allows users to quickly find different types of related resources, thereby increasing user productivity.

Bourkoukou and Achbarou^23^ aimed to build a personal recommender system that results in useful content and better recommendations in the shortest possible time^25^. The proposed system is a web-based client-side application that uses user profiles to form neighborhoods and calculates predictions using weights. To overcome the problem of a cold start, i.e. the lack of information about learners and their interests during the first communication, their profiles are created using the learning method. Resources that are of interest to the user are suggested through predictions calculated by the new functions and the collaborative filtering method. This method reduces both the problem of cold start and data sparsity.

### Machine learning-based methods

Thanh-Nhan et al.^24^ introduced a method for integrating social networks into intelligent tutoring systems (ITA) that can predict student performance^26^. To do this, the matrix factorization method is used along with social networks. One of the advantages of this work is that the relationships between students can be used to build models, thereby improving the predictive results.

Pupara et al.^25^ aimed to generate a recommendation and modeling system that uses students' characteristics and opinions to accurately predict and select the most appropriate institution for specific students through data analysis methods^27^. This system consists of three main stages of design, development, and analysis to suggest to students a suitable university. To do this, the decision tree and association rules were used, providing acceptable results for the four factors of university compulsion, trust in institutions, learner skills, and family income.

Rodríguez et al. l^12^ proposed an educational recommender system based on argumentation theory that can combine content-based, collaborative, and knowledge-based recommendation methods or act as a new recommendation method. This method provides educational objects to the student that can generate further arguments to justify their competence^14^.

### Artificial intelligence-based methods

Duque Méndez et al. l^26^ proposed a CBR-based intelligent personal assistant that can perform user-requested operations and access information from remote sources^28^. This system is a particular recommender system because of its use in web searches. Therefore, the personal assistant allows the user to interact, display, and select items according to needs and priorities. The proposed intelligent personal assistant enables users to select educational resources from learning repositories. In this regard, a recommender system has been implemented based on an artificial intelligence method called CBR. CBR is a method in artificial intelligence that tries to solve new problems like humans do, using the experiences they have gained in similar events to make decisions in similar cases^29^. In the method used, first, it is necessary to identify the elements in students' profiles and to learn object metadata. Then it is essential to define a criterion for retrieving the most similar items and specify the update of these items. One of the advantages of this article is the use of different educational resources.

Neto (2018)^30^ developed a multi-agent recommender system, which helps e-learning recommendation systems to offer students the most appropriate educational resources. This work utilizes multi-agent technology to develop a system that combines web usage and extraction algorithms, such as content-based methods and collaborative filtering to find the most appropriate training resources. The performance of this combined method is better than each algorithm.

Advances have also been made in building models for searching and retrieving learning objects stored in heterogeneous repositories. Paula Rodríguez (2013) proposes integrating two multi-agent models focused on delivering specific LO adapted to a student's profile, and delivering LO to teachers to assist them in creating courses^31^. This aims to have an integrated multi-agent model that meets the needs of students and educators and thus improves the learning and teaching process.

in^3^ a personalized education system based on hybrid intelligent recommendations. Specifically, a hybrid framework of artificial intelligence is proposed, which focuses on the way to provide targeted recommendations for the implementation of integrated standard lesson plans, which will be the main tool for creating flexible differentiated pedagogical programs that will perfectly meet the personal needs and particularities of each student.

In^5^, a multi-level methodological proposal for the automatic adaptation of open learning is presented resources, in order to provide tools that help access and properly use their metadata E-learning environments are researched with students with disabilities to determine their real needs and preferences, emphasizing the need to reinforce adequate explanations and coherent alternative text In pictures, correct subtitles in videos and audio to text conversion, data that is related to us Proposal. The purpose of the conducted research is to contribute to an automatic support tool in the production of Accessible learning resources that are properly tagged for search and reuse. This research also aims to Support researchers in AI applications to address age-old challenges and opportunities in Virtual training, in addition to providing an overview that can help training producers maintain their resources and interest in accessing them.

### Methods based on neural networks and deep learning networks

Paradarami et al. (2017)^32^ proposed a hybrid recommender system for vote prediction using an artificial neural network framework that uses both the capabilities of the content-based method and collaborative filtering to model training. This method combines content (user and business), participation (comments and votes), and vote-related metadata under a single learning model with an observer that provides better results compared to collaborative filtering recommendation systems. In this method, a multi-class classification model is developed to predict the class of a vote. One of the advantages of this is that ANN can be extended to other classes and any user in the system, which makes the model highly scalable.

Xiao Wang et al. (2017) proposed an e-learning recommendation framework based on deep learning^33^. This model is based on deep learning that can learn from large-scale data. The deep network used is GRU^34^, a type of LSTM return network. One of the advantages of this work is the use of the K-nearest neighbor method to train the model, the accuracy of which is guaranteed. Second, it can recommend new items whose similarities cannot be calculated. Third, it dramatically reduces system performance, which benefits the actual applications of recommenders.

In [54], an advanced recommendation method called AROLS has been proposed. It is integrated with a comprehensive learning model style for online students. This work suggests recommendations by considering the learning method as prior knowledge. In this way, first, it creates clusters of different learning styles; second, the behavioral patterns presented by the learning resource similarity matrix and the communication rules of each cluster are extracted using students' review history. Finally, it creates a set of personal recommendations with variable sizes based on the data mining results of the previous steps. Experiments show that the method presents the recommendations more accurately while maintaining the computational advantage over the traditional recommendation of participatory filtering (CF).

In^35^, researchers develop a recommendation mechanism for the adaptive learning system, which considers the theory of learning style by combining traditional recommendation algorithms with the clustering technique. The experiments show that the proposed method can perform better and has a computational advantage over the conventional recommendation.

In^36^, a method that integrates the features of the online learning style in the recommendation algorithm is proposed. In general, it consists of the features of participatory filtering (CF), association rules criteria (ARM), and online learning style (OLS). The proposed method has a 25% improvement over the scheme without student characteristics.

In^37^, attention concentration neural networks (CNN) have been used to collect user information, predict user rankings, and recommend top courses. In this work, the participatory filter and an attention-based CNN method are integrated to enable real-time recommendations and reduce server workload. The learner behaviors and learning history are shown as feature vectors; Also, the attention mechanism is used to improve the relationship estimation by considering the difference between the estimated and the actual scores provided by users for neural network training. Finally, the trained model recommends courses to students. However, the proposed system may suffer by recommending similar courses due to a large number of them with the development of MOOCs. This module shows the type of learning and learning habits of the user, such as the time of study per week, dropout rate, etc.

With the rapid rise of MOOC platforms, online learning resources are on the rise. Since learners differ in their ability to recognize and structure knowledge, they cannot quickly identify the learning resources they are interested in. Traditional technologies that recommend collaborative filtering work very poorly and have problems such as data sparsity and cold start. In addition, duplicate recommended content and high-dimensional, non-linear data on online learning users cannot be effectively managed, leading to resource recommender inefficiencies. To increase learner productivity and enthusiasm, Zhang et al. (2018) introduced a highly accurate resource recommendation (MOOCRC) model based on deep belief networks (DBNs) in MOOC environments^33^.

This method deeply extracts the characteristics of the learners and their lesson content and incorporates the behavioral characteristics of the learners to construct the vector of user-lesson characteristics as the input of the deep model. Learners' scores on lessons are processed as supervised learning tags by the supervisor. The MOOCRC model is trained with supervisors without pre-training and is fine-tuned using supervisor feedback. In addition, this model is obtained by repeatedly adjusting the model parameters. To evaluate the effectiveness of MOOCRC, an experimental analysis is performed using selective data from learners obtained from the starC MOOC platform of Central China Normal University. Learners' actual participation data in lessons are used to assess the accuracy of the MOOCRC classification. The results show that MOOCRC has higher recommendation accuracy and faster convergence than other traditional recommender methods.

In the following, some specialized concepts will be stated, and in the next section, the details of the proposed method will be explained, which is a combined architecture of deep learning networks with compression technique and separation of activity time into two short-term and long-term. In the fourth section, the results of the implementation of the proposed algorithm will be reviewed, and finally, in the fifth section, the content will be summarized, and suggestions will be presented.

In^13^, we have used the resource advisor system as an educational environment to recommend educational resources to students so that these recommendations are tailored to the preferences and needs of each student. We present the resource recommending system as a combination of improved deep learning networks MLP, BiLSTM, and LSTM using the attention method. Compared to similar studies using DBN networks and focusing only on the interests and preferences of users in the recent previous, the proposed system, in addition to the previous long-term interests of the user, offers higher accuracy and more appropriate recommendations according to current interests.

## Conclusion

Different techniques and methods have studied recommender systems and educational recommender systems. Most of these researches, especially regarding an educational recommender for recommending scientific resources, have tried to solve the problem with linear and data mining methods and models such as ontology^7,15,17,19,23,38–41^.

A large amount of information is one of the problems and limitations of these methods; they only introduce a framework for recommendations. An educational consultant that automatically provides useful advice is not much discussed. Some others have also benefited from machine learning and artificial intelligence methods, which have shown a more suitable efficiency for solving problems than the previous methods^15,23,39^. One of the best ways to solve the problem is to use the structure of artificial neural networks^30–32,42^. Continuing the evolution of neural networks, deep learning networks are one of the newest and most complete solutions.

These types of networks can solve problems with high accuracy due to the acceptance of a massive amount of problem data, neural network integration, learning techniques, and structural dynamics in forming the number of hidden layers. The issue of educational advisors was not exempted from this issue, and Most of the news articles presented in the field of recommendation have used this technique. It can be claimed that today's topic and solution in the field of recommenders is deep learning techniques and their combination models, along with the use of large-scale data (big data).

But in many of these methods, the exact weight is considered for all the users' interests in learning. Only the user's previous information is used for education. While in the present Article, having a network that looks both backward and forwards, it is possible to cover the changes in the learner's behavior and suggest more updated recommendations.

Previously, short-term and long-term user interests were not used in educational recommenders, and this structure in the proposed model of this article was able to cover the dynamics of users' interests and needs. On the other hand, in this article, by using deep learning networks, we have been able to use the large volume of available datasets with the effectiveness of the time of occurrence for model training.

## Proposed method

The implementation process of our proposed recommender system includes 5 phases of data mining from the OULSD standard database, data preprocessing, and model construction with a combination of deep learning networks such as LSTM, MLP, GRU, Bilstm improved with attention technique^43,44^, weighting parameters, and training. Finally, using the trained network, we recommend resources to users.

**Figure 1:**
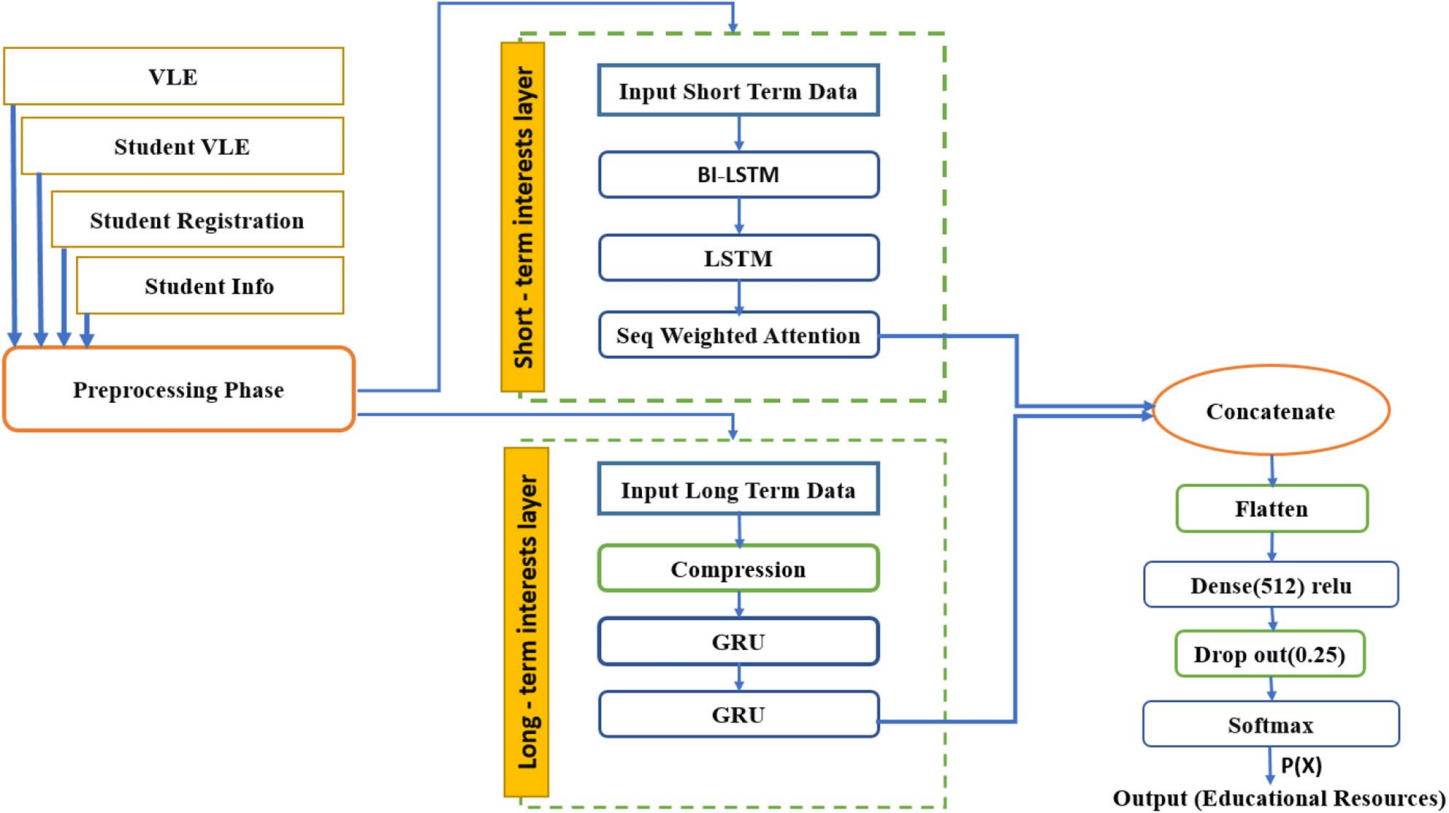
Proposed network architecture.

The proposed model Fig. 1 consists of two independent blocks for processing long-term interests and short-term interests.

The goal is to focus on feature extraction. We want to extract features in two different levels. We extract the interests and characteristics of the user in the short term and the long term separately. In fact, the change in user behavior can be found from long term to short term. The model learns to have better behavior and recommendations in relation to users whose interests change over time. Two independent blocks in the proposed architecture, after combining them together, try to extract such variable behavior patterns in users. Dataset records are divided into short-term and long-term sections based on a specific time axis. This separation of user interests and different views and valuing of interests based on the time axis, while increasing the system's accuracy and not encountering too many errors, suggest suitable and personalized resources based on the needs and tastes of students. Short-term interests play a more effective role than long-term interests in offering educational resources.

Researchers have looked at short-term interests as a fixed feature in many previous works and therefore assigned the same weight to the items. Then the compression algorithm presented in this article is applied to the long-term part, and the data is compressed in both row and column dimensions. In the following, for the users in the data bank who have not been active in a short period, we have considered their last activity in the long-term sector as the activity in the short-term sector. After the design and creation of the model, the training begins. At this stage, for each user, the first record of his activity in the short-term section is repeated for all his activities in the long-term section (this work is repeated for the number of records in the short-term section).

Finally, the remaining records from the database have been used to test the loss and accuracy of the test data set.

### Data preprocessing

**Figure 2:**
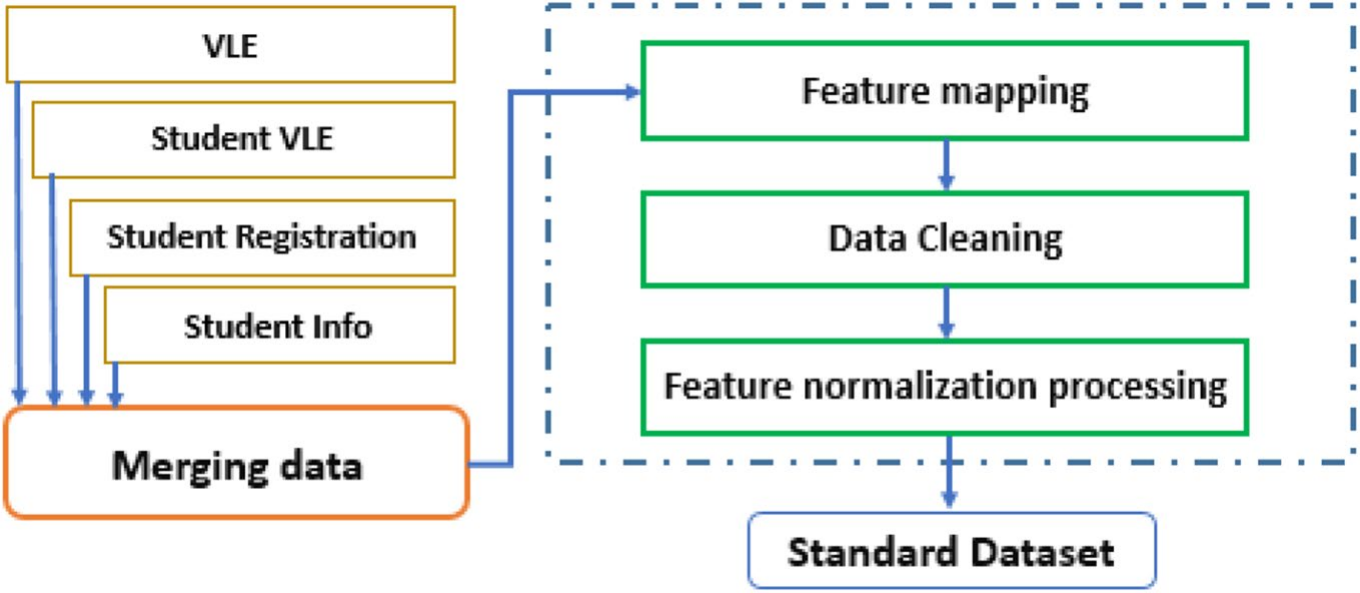
Data Preprocessing Steps.

The steps in the preprocessing can be seen in Fig. 2. First, we extract the provided resources, student features, courses held, and student performance and evaluation in each course from the OULAD standard database. After merging the data, we proceed to categorize and map the features by Converting the string values of the quantitative variables in the database to numeric values, deleting the empty or incorrect data, and normalizing the features.

After merging the data with Formula(1), we proceed to normalize the feature in the domain [0,1].

1 $${x}^{*}=\frac{x-{x}_{min}}{{x}_{max}-{x}_{min}}$$

where x_min_ represents the minimum, x_max_ is the maximum value, x* is the normalized value, and *x* is the original data.

**Dataset** (https://analyse.kmi.open.ac.uk/open_dataset) As input to the training and testing phase of our proposed model, we have used the standard analytical learning database of OULAD Free University, which is stored in CSV format^45,46^.

This database is collected from sample data from students, including demographic data, the courses attended, their set of study activities during the course, and the final results of each course. In detail, it contains the students' interactions with Virtual Learning Environment (VLE) for seven selected courses. The dataset includes 22 modules of over 30,000 students. The data is fetched by the daily summaries of student clicks on several resources. In OULAD, the tables are connected using unique identifiers; The number of data records used in this article after the initial stages of pre-processing is 10,403,715 and each record consists of 12 fields. Due to the large amount of data and the tendency to influence time in the model training process, it will be useful to use deep learning methods to solve the problem raised in the article. It should be noted, that the tables are stored in the CSV format. The utilized files are briefly described below:

- **Assessments:** These contain information about assessments in module presentations.
- **Student Info:** This file holds data about students' demographics along with their results. In addition, each student can have several records.
- **Student Vle:** Includes students’ clicks and interactions with resources available in Vlr that can be in Html, pdf, etc. formats.
- **Student Assessment:** keeps the results of the evaluations made during the course per student
- **Student Registration:** This table encompasses information about the student registration time to participate in the module. Besides, it is recorded for students who have not registered the registration date.
- **Vle:** Contains information about the tools existence in the VLE. It is usually html, pdf, etc. pages. Students have access to these resources online and their interactions with them are then recorded.

Table 1, shows an example of the values available in the original database (OULAD)^46^ that are mapped to numerical values in Table 2 (designed by the researcher).

**Table 1:** A sample of database data before mapping.

Code_module	Code_presentation	Id_student	Gender	Highest_education	Age_band	Final_result	Score_mean	Id_site	Date	Sum_click
AAA	2013J	11391	M	HE qualification	55<=	Pass	82	546,669	-5	16
AAA	2013J	11391	M	HE qualification	55<=	Pass	82	546,662	-5	44
AAA	2013J	11391	M	HE qualification	55<=	Pass	82	546,652	-5	1
AAA	2013J	11391	M	HE qualification	55<=	Pass	82	546,668	-5	2
AAA	2013J	11391	M	HE qualification	55<=	Pass	82	546,652	-5	1
AAA	2013J	11391	M	HE qualification	55<=	Pass	82	546,670	-7	2
AAA	2013J	11391	M	HE qualification	55<=	Pass	82	546,671	-7	2

**Table 2:** The values of features mapped to the number.

Code_module		Code_presentation		Age_band		Gender		Highest_education		Final_result	
AAA	0.1	2013B	540	0–35	0.1	F	0.1	A Level or equivalent	0.1	Distinction	0.1
BBB	0.2	2013J	720	35–55	0.2	M	0.2	HE qualification	0.2	Fail	0.2
CCC	0.3	2014B	180	55<=	0.3	–	–	Lower than a level	0.3	Pass	0.3
DDD	0.4	2014J	360	–	–	–	–	No formal quals	0.4	Withdrawn	0.4
EEE	0.5	–	–	–	–	–	–	Post graduate qualification	0.5	–	–
FFF	0.6	–	–	–	–	–	–	–	–	–	–
GGG	0.7	–	–	–	–	–	–	–	–	–	–

### Records labeling

Since the available data do not have a defined label, in this research, we have labeled the records as follows:

From the set of activities recorded for each student's joint courses, from the course that has the highest score in his assessments (which can be the effect of the resources studied), the source that has the most clicks (which can indicate the taste and interest of the student) has been chosen as the label.

**Figure 3:**
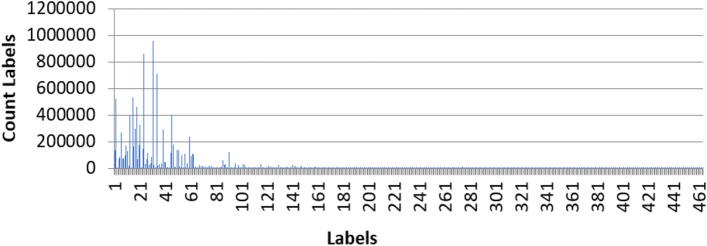
Frequency of count labels (Method 1: The most click in the maximum average score).

The result of data labeling was the separation of sources into 562 categories. Their frequency can be seen in Fig. 3. At the end of the preprocessing phase, the data is divided into training, test, and validation sets.

### Investigating the correlation between variables and labels

**Figure 4:**
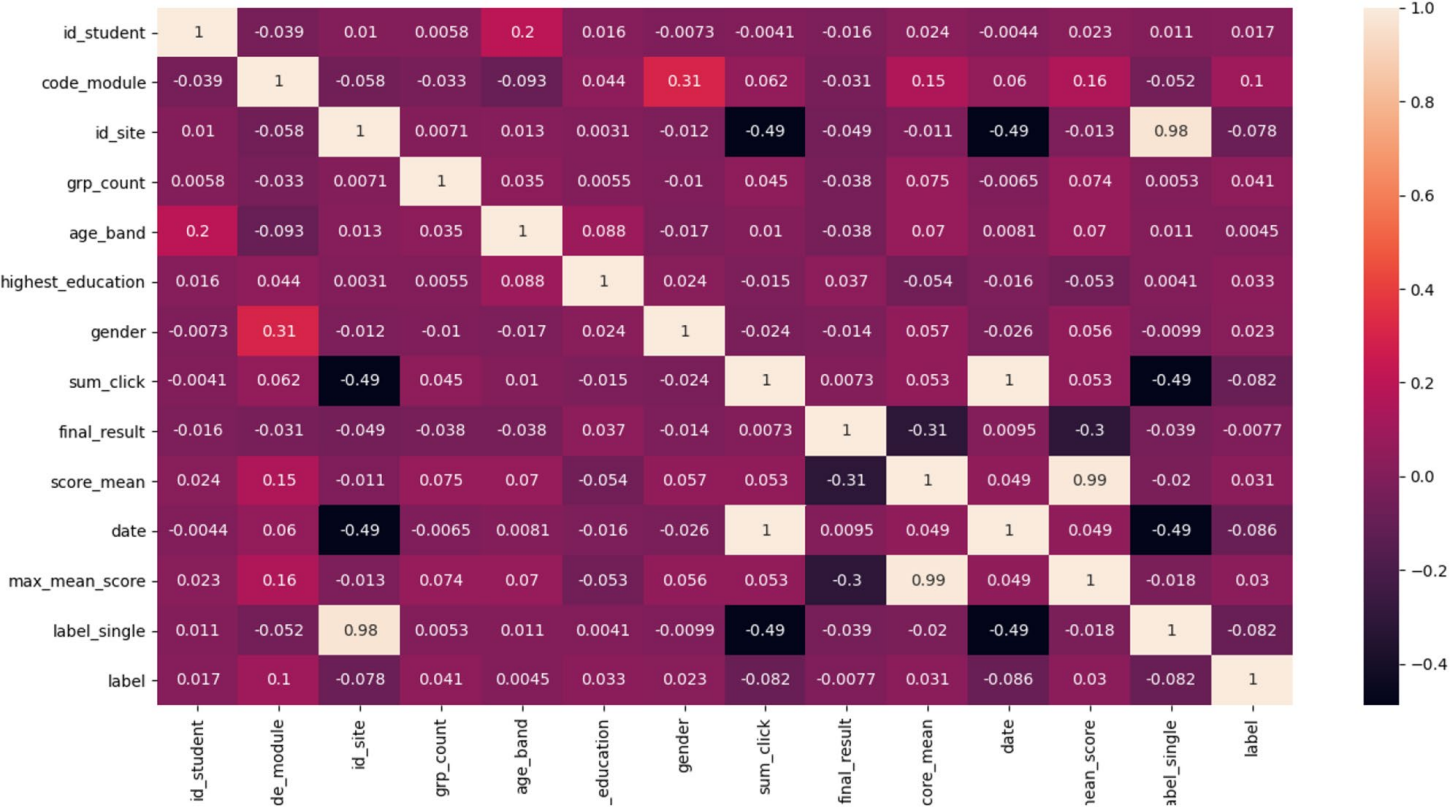
Correlation test result.

To investigate the possible correlation between the label and the existing variables that were used as input to train the model, we used the correlation test, and as you can see in Figure Fig. 4, there is no significant correlation between the variables and their label.

### Symbols

In the recommendation system, one aspect of the choice is the user and the other vital aspect is the selected option, which could be a course by a student, a movie by a viewer, a track of music for a listener, or a meal by an eater and so on^36^.

Assuming U represents a collection of users and I is the items chosen by the user. In this scheme, the main goal is extracting the interests and priorities of users by looking at User-Item interactive events. For example, clicking on an educational source is considered an action for users. Also, for each user, u ∈ U is a sequential time window as ${W}_{u}=\{{w}_{1}u, {w}_{2}u,{w}_{3}u, \dots ,{w}_{t}u\}$ which t represents the total number of time windows; Also, ${w}_{t}u$ shows a collection of smaller time units as ${{\text{w}}}_{t}u=\left\{{d}_{1}, {d}_{2},\dots ,{d}_{x}\right\}$ where x indicates the length of time windows. Moreover, the related items of the user (*u*) in time windows (t) express by ${{\text{w}}}_{t}u$. There are some events in each time window as $\{et, iu\in Rm |{\text{i}}=1, 2, ...,\mathrm{ t}$ } that iu and et describe the event* i* in time units of windows time (*d*_*x*_). Something else which should be mentioned is that user u interacts with the item |$i$∈I| in each event^36^.

For a time stage t, the S_t_^u^ session represents the short-term interests of the user at time t, and the sessions before the time stage t represent the long-term interests of the user, which is defined as L_t−1_^u^ = S_1_^u^ ∪ S_2_^u^ ∪ … ∪ S_t−1_^u^. Our goal is to predict the next learning resource (e_t,i+1_^u^) in the S_t_^u^ session.

In this scheme, the time axis that determines short-term interests and long-term interests is considered to be the last 6 months, which is equivalent to half an academic year. This means that user-item interactive events in the last six months of the user are in the short-term category, and other interactions since the user's birth are in the long-term category.

The data in the train set are divided into two categories: short-term interest data and long-term interest data, and then the resource recommendation is generated as the output of the model. After training the model to reach a certain error value, the test set can be used to test the efficiency of the recommendation model.

#### Short-term interests section

Researchers have looked at short-term interests as a fixed feature in many previous works and therefore assigned the same weight to the items. As a result, diversity in short-term interests has not been properly evaluated. To recommend the next source, the user's short-term interests are essential. Thus, the proposed model's architecture based on the attention technique has given weight to both long-term and short-term sessions. With this technique, the characteristics of the user u are fully taken into account. In addition, two-way LSTM is used to predict the recommendation to look at both the previous and the future and be sensitive to the variation in the user's short-term interests in both directions; this way, learners' behavioral changes can be covered.

To discover features and learners' behavioral changes, we propose a module based on two-way LSTM networks to extract periodic features and capture such time dependence of the input feature. In this research, we have evaluated single-layer and double-layer models.

The input with length 12, ${\text{I}}=\{{i}_{1},{i}_{2},\dots {i}_{12}\}$ is entered into the proposed model and the output ${y}_{t}$ is calculated according to formula (2).

$${h}_{t}^{f}={\text{tanh}}({W}_{xh}^{f}{x}_{t}+{W}_{hh}^{f}{h}_{t-1}^{f}+{b}_{h}^{f})$$

2 $${h}_{t}^{b}={\text{tanh}}\left({W}_{xh}^{b}{x}_{t}+{W}_{hh}^{b}{h}_{t+1}^{b}+{b}_{h}^{b}\right)$$

$${y}_{t}={W}_{hy}^{f}{h}_{t}^{f}+{W}_{hy}^{b}{h}_{t}^{b}+{b}_{y}$$

As you will see in Table 6, the results obtained from the two-layer BiLSTM network, where the output ${y}_{t}$ is passed from the lower layer to the input of the upper layer, are more favorable.

The main idea of the attention technique is to learn to assign accurate (normalized) weights to a set of features. So, higher weights indicate that the corresponding feature contains more important information for the given taskFig. 5.

**Figure 5:**
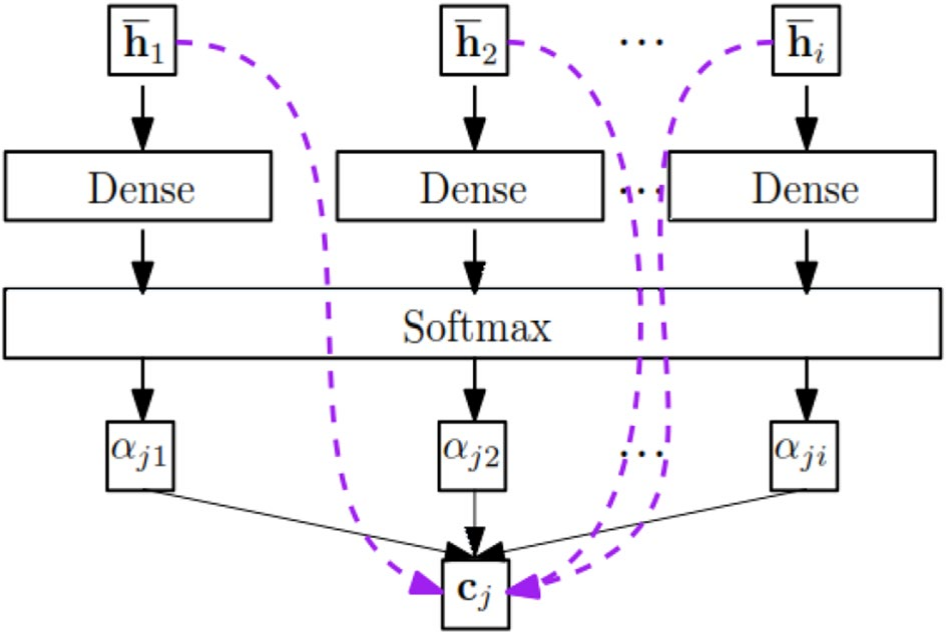
The block diagram of the proposed method.

Attention techniques are divided into two categories based on calculating attention scores.

(1) Standard vanilla attention and (2) Collaborative attention. Note Vanilla uses a parameterized content vector, while collaborative attention is related to learning attention weights from two sequences. In this research, method 1 (formula (3)^47^) is used.

$${u}_{t}={\text{tanh}}({\text{W}}{\hat{h} }_{t}+{\text{b}})$$

3 $${\alpha }_{t}= \frac{{\text{exp}}({{\text{u}}}_{t}^{T}u)}{\sum_{t}{\text{exp}}({{\text{u}}}_{t}^{T}u)}$$

$$v=\sum_{t}{a}_{t}{\hat{h} }_{t}$$

${u}_{t}$: The vector of valuing the features.

${\alpha }_{t}:$ Normalized weight of features obtained by softmax function.

$v$: The sum of all input information that includes the sum of the weights of each ${h}_{t}$ With ${\alpha }_{t}$ as the corresponding weights.

Then the v vector is entered into a fully connected layer with softmax activation to perform the final classification. The recommendation is a vector $y\in {R}^{2}$ with significant and non-significant probability. Using argmax, we select the highest probability as the model recommendation.

#### Long-term interests section

**Figure 6:**
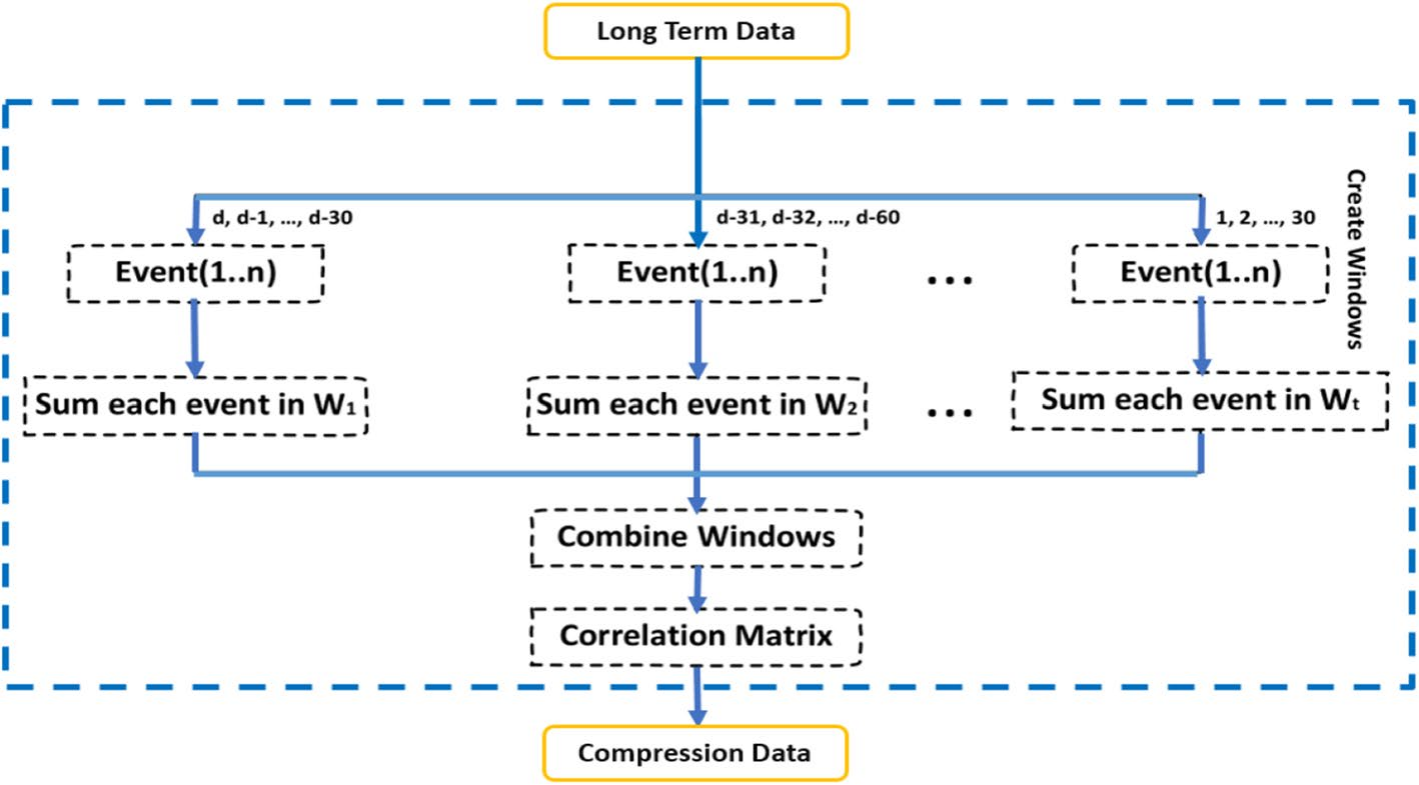
The block diagram of the proposed method^36^.

In the long-term interests section, due to the large volume of data records belonging to each student, we use the compression technique (Fig. 6) in both row and column dimensions ^36^.These data are entered into the system to display the interests and preferences of the user in the time period of his birth to the time axis of the short-term class, which is considered in this research, the last 6 months. The used compression algorithm weights resources in different time windows with a policy.

##### Compression in record level

First, one window (or limited number) is considered as a background. In this way, the user’s different activities appearing in a time window are observed. the different weights based on the average of constituent days are assigned for each window. With the help of these techniques, each user's activity with attention to the category of the time window is marked by proportional weight. Hence, the smaller weights can be considered for distant time windows. On the contrary, due to the crucial role of near-time windows, the bigger weights are selected for them. In the following, the weight of the feature is multiplied by the summation of the corresponding activity; Then, the outcome is aggregated with the results of the rest of the windows. Finally, each feature that is considered as a background is repeated only once in the section; and in the new field, it maintains the frequency of the merged features^36^.

##### Compression in feature level

After compression at the record level, the feature selection with the correlation matrix Pearson [Formula (2)] which measures linear dependence between two variables^48^ is applied to further compress data at the feature level. After implementing the Correlation Matrix with Pearson, we get a matrix n • n, with n being the number of features. Matrix values are the Correlation Coefficient values ranging from − 1.0 to + 1.0 with − 1.0 being a total negative correlation, 0.0 being no correlation and + 1.0 being a total positive correlation. With the help of this strategy, the feature pairs with the highest correlation value are selected^36^.

After applying compression on long-term data with a window length of 7, the available data will decrease from 12*10,543,682 to 11*5,167,599, and GRU is entered as input to a two-layer multi-cell network the output ${y}_{t}$ is calculated according to formula (4).

$${z}_{t}=\upsigma ({W}_{z}\cdot [{h}_{t-1,}{x}_{t}])$$

$${r}_{t}=\upsigma ({W}_{r}\cdot [{h}_{t-1,}{x}_{t}])$$

4 $${\hat{h} }_{t}={\text{tanh}}\left(W\cdot \left[{r}_{t}*{h}_{t-1,}{x}_{t}\right]\right)$$

$${h}_{t}=\left(1-{z}_{t}\right)* {h}_{t-1}{+z}_{t}* {\hat{h} }_{t}$$

In the proposed model, to achieve better results in feature extraction, model parameters in each layer have been adjusted many times and the model has been trained, tested and evaluated.

The output from the training of short-term and long-term parts is connected, then it enters the MLP layer with a learning rate of 0.0001 and relu activation function. After applying dropout = 0.25 to avoid the effects of fully connected layers, preventing overfitting of the network in the last step enters the MLP layer with the softmax activation function. Finally, it provides one or more appropriate educational resources as output to the user. The cost function used in this structure is categorical cross-entropy. In the model training process, due to the large volume of input data, we used the minibatch method or size 1028.

In similar problems where the number of available classes is more than two classes, Softmax activation function and mutual entropy cost function are used in the output layer of the model.

## Methods and tools of data analysis

We seek to predict the best educational resources to evaluate the performance of the proposed method.

A set of criteria are tested and evaluated as follows^49^

5 $$Accuracy=\frac{ TP+TN}{TP+{\text{T}}N+FP+FN}$$

Indicates what percentage of experimental records are properly categorized.

6 $$Precision=\frac{\sum_{x\in X}|R\left(x\right)\cap H\left(x\right)|}{\sum_{x\in X}|R\left(x\right)|}$$

7 $$Recall=\frac{\sum_{x\in X}|R\left(x\right)\cap H\left(x\right)|}{\sum_{x\in X}|H\left(x\right)|}$$

8 $$F1=\frac{2*Precision* Recall}{Precision+Recall}$$

Here x is a student from the set of all students X, R (x) represents the learning resources recommended for student x, and H (x) represents the learning resources observed by learner x.^36^.

## Result and discussion

The available data have been used as network input in short-term and long-term sections. The short-term part contains 139968 records, and the long-term part contains 5,167,599 records after compression. can see in Table 3, To check the power of generalization, we have performed 4 different steps, and all cases show validation-split = 0.2 as a result. After the completion of the Epochs, as shown in Tables 12 and 13, our model has better suggestions for scientific resources due to the optimal structure in comparison with other methods.

**Table 3:** Model Test Result.

Train test split	Train 70 % Test 30 %	Train 80 % Test 20 %	Train 90 % Test 10 %	Train 99 % Test 1 %
Accuracy test	9979.0	0.9978	0.9977	0.9977

### Investigating the effect of the number of cells in each layer

Our input data is tabular; usually, a variety of long-term recursive networks converge to this type of data and texts earlier. Besides, the temporal nature of learning requires adopting methods that can use a period. So, in the first step, we implemented three types of LSTM, Gru, and Bilstm networks with single-cell structures in three single-layer, two-layers, and three-layer architectures. The aim is to investigate the effect of the number of layers in the structure of the recommender model. As you can see in Table 4, the results are not desirable. None of the 9 single-cell architectures has been able to find the pattern and the relationship between different features and their relationship in combination and fitting with different models. Even increasing the number of layers did not play an essential role in improving the results, and the decrease in the accuracy of the 3-layered model compared to the single-layered model shows that the model is moving toward overfitting.

**Table 4:** Results of training and testing of one to three-layer single-cell networks: LSTM, Bilstm, and GRU.

Model	Layer	Train		Test
		Accuracy	Val_ accuracy	Accuracy
lstm	1	0.2825	0.2879	0.28
	2	0.2607	0.2577	0.18
	3	0.266	0.2548	0.25
GRU	1	0.2389	0.2539	0.25
	2	0.2439	0.2562	0.25
	3	0.2317	0.2388	0.23
Bilstm	1	0.4481	0.6298	0.62
	2	0.3133	0.3231	0.31
	3	0.3057	0.3132	0.31

In the second step, we achieved better results by increasing the number of cells in each layer, equivalent to the number of features available as model input. As seen in Table 5, the multi-cellular single-layer architecture implemented and studied in three single-layer LSTM, Bilstm, and Gru architectures has better results than single-cell architecture. Meanwhile, the Gru network with higher generalizability, an Loss of 0.2, and an accuracy of 0.91 has better performance than the two other types of networks.

**Table 5:** Results of training and testing single-layer multi-cellular networks of LSTM, GRU, and BILSTM.

Model	Train		Test
	Accuracy	Val_ accuracy	Accuracy
LSTM	0.7573	0.7704	0.77
BiLSTM	0.8757	0.9036	0.90
GRU	0.8980	0.9099	0.91

### Investigating the effect of the number of layers of network architecture

To evaluate the effect of increasing the number of layers, we have implemented and trained three two-layer multi-cellular architectures with LSTM, Gru, and Bilstm networks. The results in Table 6 show that two-layer architectures have been better than single-layer architectures. Also, they have been more successful in finding the pattern and the relationship of different features.

**Table 6:** Results of training and testing of two-layer LSTM, GRU, and BILSTM networks.

Model	Tain		Test
	Accuracy	Val_ accuracy	Accuracy
LSTM	0.9021	0.9169	0.91
GRU	0.9013	0.9121	0.92
BiLSTM	0.9529	0.9539	0.95

### Investigating the effect of using the attention mechanism

The method of "attention" is derived from human visual attention. Just as man focuses on certain parts of visual inputs for cognition or perception. Integrating the "attention" technique with Rnns helps to process long and noisy inputs. Although LSTM can theoretically solve the problem of long memory, it still has problems when faced with long intervals. The attention-based network technique helps in remembering long-term inputs. Using the attention technique in recommender systems removes useless content while maintaining interpretability and selects the items with the most representation^46^]. We have evaluated the attention technique's effectiveness by implementing single-layer multi-cellular Lstm, and Bilstm networks. As seen in Table 7, the use of attention techniques in network architecture has positively affected the results.

**Table 7:** Results of educating and testing of LSTM and BiLSTM network and the attention technique.

Model	Train		Test
	Accuracy	Val_ accuracy	Accuracy
LSTM and attention techniques	0.9423	0.9366	0.94
GRU and attention techniques	0.949	0.9331	0.94
BiLSTM and attention techniques	0.7458	0.7580	0.70

The reason for the failure of the single Bilstm layer with the technique of paying attention to the proportion of the single Bilstm layer can be a large number of output neurons of the Bilstm layer. When Bilstm is used in a model, the output of two Lstm layers is actually stitched together and the number of generated features is doubled. Since the failure to extract features when faced with very long sequences has been one of the weaknesses of basic attention. This means that they have problems extracting the global features and only succeed in extracting local features. In the results of Table 7, you can see that the layer combined with the attention technique was weaker than in the case where the attention technique was not used.

### Compression evaluation

To evaluate the compression method, we have trained and evaluated five architectures: LSTM, GRU, LSTM + Attention, GRU + Attention, and Bilstm in 3 different window lengths. Researchers sometimes miss network training due to a large amount of data and the lack of access to appropriate hardware. As shown in Table 8, this is possible by selecting the appropriate window length and designing the application model. We have trained and tested original and uncompressed data in 50 courses for different architectures. We have trained and tested similar architectures with 100 courses during 7, 14, and 30-day windows. In this way, you can see that in addition to using fewer hardware resources such as RAM, despite doubling the number of courses, a lot of time is saved.

**Table 8:** Investigating the effect of selected window length in accuracy and loss of training and testing phases.

Dataset	Percentage reduction of data records after compression	Average execution time in Colab for trained models (seconds)	Average execution time in PC for trained architectures (seconds)	Average accuracy in trained models
Original	0	NAN	84657.2	0.88
Compressing (WI=7)	50.98867%	22504	49875.5	0.88
Compressing (WI=14)	59.78858%	15982	42604.1	0.78
Compressing (30)	67.78494%	13383.9	33071.8	0.682

As you can see in Table 9, by comparing the results of the original data and the accuracy and loss after data compression in the training and testing phase of the implemented models, the network with window lengths of 7 and 14 Learns with high speed and acceptable accuracy. As can be seen (Table 9), in the window with a time duration of 7, the average accuracy of training and testing of implemented models is maintained despite having the data and increasing the execution speed. Also, for a window with a length of 14, when our data volume has reached approximately 40, the accuracy has decreased by 0.10%.

**Table 9:** Summarizing the impact of compression in terms of accuracy, saving memory and time.

Structure of network		Original dataset (epoch 50)				Compress Win 7 ( Epoch 100)			
		Time (seconds)		Accuracy		Time (seconds)		Accuracy	
		PC	Colab	Val	Train	Colab	PC	Val	Train
Lstm	Train	115,395	Low Memory or Time	0.8652	0.8645	15,853	65,003	0.8767	0.8872
	Test	1933		**0.90**		686	983	**0.88**	
Gru	Train	154,456		0.8809	0.893	42,546	82,781	0.9048	0.9087
	Test	2486		**0.89**		836	1558	**0.91**	
Lstm + Attention	Train	172,034		0.9122	0.9229	15,108	90,539	0.8855	0.8934
	Test	1970		**0.94**		720	1056	**0.89**	
Gru + Attention	Train	205,520		0.809	0.815	48,011	93,042	0.8977	0.9034
	Test	2518		**0.81**		1005	1756	**0.90**	
Bilstm	Train	188,240		0.8299	0.8609	99,400	160,885	0.8104	0.8256
	Test	2020		**0.86**		875	1152	**0.82**	

Structure of Network		Compress Win 14( Epoch 100)				Compress Win 30( Epoch 100)			
		Time ( seconds)		Accuracy		Time ( seconds)		Accuracy	
		PC	Colab	Val	Train	Colab	PC	Val	Train
Lstm	Train	53,769	12,529	0.7506	0.7735	10,418	43,670	0.5592	0.5986
	Test	762	587	**0.77**		296	556	**0.60**	
Gru	Train	68,944	30,388	0.837	0.8508	25,549	55,505		
	Test	1347	583	**0.85**		311	1153	**0.72**	
Lstm + Attention	Train	86,345	11,982	0.7864	0.8029	9816	59,918	0.6811	0.7219
	Test	839	654	**0.80**		412	702	**0.72**	
Gru + Attention	Train	79,475	42,066	0.7911	0.8001	35,313	60,301	0.7283	0.753
	Test	1520	875	**0.80**		401	1293	**0.75**	
Bilstm	Train	132,128	59,400	0.6723	0.686	50,733	106,813	0.5721	0.6218
	Test	912	756	**0.69**		590	807	**0.62**	

Wi means the length of the window.

Significance values are in bold.

According to Table 10, the results of our proposed architecture, with a loss rate of 0.005 and an accuracy of 0.997, are much more accurate and desirable than the proposed architecture^46–51^. Our proposed architecture has two short-term and long-term compressed layers with a window length of 7 and uses an improved two-sided bilstm structure with attention and GRU technique, which according to Table 8, has good generalizability. We also trained and tested our proposed architecture on compressed data with different window lengths, and the results (Table 10) were also very desirable with high window length compressions.

**Table 10:** The result of the proposed network in terms of train and test accuracy.

	Train		Test
Win7	Accuracy	Val_ accuracy	Accuracy
	0.9969	0.9977	0.998
Win14	Accuracy	val_ accuracy	Accuracy
	0.9901	0.9939	0.994
Win30	Accuracy	val_ accuracy	Accuracy
	0.9825	0.9914	0.99
Win60	Accuracy	val_ accuracy	Accuracy
	0.9664	0.9812	0.98

**Figure 7:**
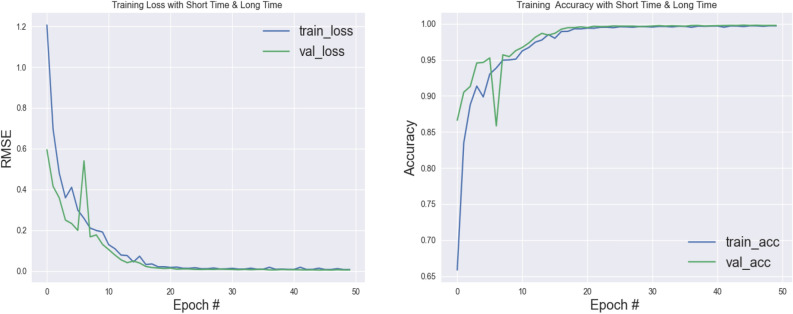
Proposed network accuracy and loss diagram (data compression with window length 7).

As can be seen in Fig. 7, the loss rate at the final AIPACs indicates that the number of selected AIPACs is appropriate, and since the validation and validation accuracy diagrams are approximately the same, it is clear that no overfitting occurred in this experiment. The decreasing slope of the loss charts in the early AIPACs also indicates the appropriateness of the learning rate selected in our training process.

### 5-fold cross-validation

Mutual validation is a model evaluation method that determines the extent to which statistical analysis results on a data set can be generalized and independent of educational data^47^. The data is divided into five subsets, each used for validation and the other 4 for training. This procedure is repeated 5 times, and finally, the average of the results is chosen as a final estimate. The results of the validation of the proposed model are shown in Table 11.

**Table 11:** Results of the K-fold method on data accuracy.

5-Fold	K=1	K=2	K=3	K=4	K=5
Accuracy	99.57	99.89	99.71	99.78	99.82

### Comparison of the performance of the proposed model with other models

We have compared the results of the proposed model in the first row of Table 12 with other methods presented in related work or implemented by ourselves. As can be seen, the results are more desirable for different evaluation parameters of the proposed model than other implemented methods. All evaluations were performed on OULAD-shared data.

**Table 12:** Comparison of the proposed method with the results obtained from other implementations performed by us and studies^37,50–52^.

Model	Accuracy	Recall	Prec	F1	Ref
Proposed model	0.9978	0.8898	0.8826	0.8791	–
KNN baseline (knn)	0.9865	0.9295	0.9865	0.9864	
Naive bayes(nb)	0.1981	0.4725	0.2937	0.1853	
Logistic regression(Lr)	0	0	0	0	
Latent dirichlet allocation(lda)	0.5415	0.0772	0.4314	0.4579	
SVD	0.2281	0.4975	0.3231	0.2157	
Bi-LSTM + LSTM + attention	0.967	0.9698	0.9576	0.9636	^53^
DBN	0.2912	–	–	–	^50^
AROLS	–	0.3	0.29	0.55	^37^
itemCF	–	0.5	0.18	0.57	
Clustering + itemCF	–	0.85	0.24	0.86	
ItemCF	–	0.026	0.1334	0.0435	^51^
Item-AROLS	–	0.0406	0.1880	0.0668	
User-AROLS	–	0.0018	0.0046	0.0026	
UserCF	–	0.0005	0.0011	0.0007	
CF with ARM	–	0.6874	0.076	0.1374	^52^
Method article	–	0.8647	0.1033	0.1842	

The proposed method^50^ has been implemented, trained, tested, and evaluated with OULAD data. As shown in Table 12, it performed worse than our proposed model in terms of both Loss and accuracy criteria.

In^37^, the three criteria, including Recall, Prec, and F1 for the three methods itemCF, Clustering + itemCF, and AROLS are examined. It shows that the proposed algorithm (AROLS) has a better Prec than the other two cases. Meanwhile, F1 and Recalls remain relatively steady at n top recommendation simultaneously.

The work^51^ shows that AROLS performs much better than traditional participatory filtering, especially User-AROLS calling, and accuracy, which has more than tripled. Also, the calling accuracy of UserCF is much smaller than ItemCF, probably because UserCF focuses more on the interests of learners who are more like a particular learner. At the same time, the ItemCF recommendation is more personal because it primarily suggests similar items based on the learner's interest. As can be seen in the first row, the proposed model performed better than all seven reviewed methods.

In^52^, the results show that OLS characters can make the recommendation algorithm more accurate and robust, but as seen in the first row, the proposed model performed better than both studied methods.

### Investigating the scalability and time complexity of the proposed method

We have trained and tested the proposed model with different data volumes to check the scalability and time complexity. The results in Table 13 show that reducing the volume of input data reduces time complexity, but the accuracy obtained is still desirable, indicating our proposed model's scalability.

**Table 13:** Investigation of scalability and time complexity of the proposed method.

Number of records	Train time(sec)	Accuracy	Val_accuracy	Accuracy-test
Win7 = 5,167,599	133,394	0.9972	0.9981	0.997
Win14 = 4,239,764	106,375	0.9901	0.9939	0.99
Win30 = 3,396,653	87,024	0.9825	0.9914	0.98
Win60 = 2,816,927	72,806	0.9664	0.9812	0.966
1,816,927	46,554	0.9328	0.9563	0.93
816,927	21,529	0.7371	0.7722	0.73

## Conclusion and future works

Recommender systems, especially educational recommender systems, have been studied using different techniques and methods mentioned in the previous sections. Most of these studies, especially in the issue of the educational recommender to recommend scientific resources, have tried to solve the problem with linear methods and models and data mining such as ontology^19–26,28^. One of the problems and limitations of these methods is that they do not accept a large amount of information, and in some of them, only a framework for recommendation is introduced, and the training recommender who can automatically suggest useful advice has not been much discussed. Others have used machine learning methods and artificial intelligence and such techniques have shown better efficiency in solving the problem than previous methods^15,29–34^.

One of the best methods to solve the problem is to use the structure of artificial neural networks^21,35–37^. Following the evolution of neural networks, one of the newest and most complete solutions is the use of deep learning networks. These types of networks can solve problems with high accuracy due to the receptivity of a large amount of problem data, integration of neural networks, learning techniques, and structural dynamics in the formation of several hidden layers. The issue of the educational recommender is no exception to this case; most of the news articles presented in the field of recommendation have used this technique^21,43,45^. It can be claimed that deep learning techniques and their hybrid models and big-scale data (bulk data) are the topic and solution of the day in the field of recommenders.

However, in many of these methods, the same weight is considered in learning for all users' interests, and only the user's previous information is used in learning. While in the present dissertation, having a network that looks both backward and forwards, it is possible to cover changes in learner behavior and offer more up-to-date recommendations.

Due to the advancement of science and the production of new scientific resources, educational resources are constantly increasing, and the network needs to be trained permanently and gradually. However, in most systems, network learning is based on existing resources and does not look to future resources. As a result, due to the rapid growth of these educational resources, network offers will soon become unusable and outdated. This challenge can also be addressed by graduating from the network training process. However, given the following problems, more work needs to be done in the future to achieve effective recommendations. These problems include (1) incremental learning for non-stationary and current data such as large user volumes and input items, (2) computational efficiency for high-dimensional tensors and multimedia data sources 3) balancing model complexity and scalability despite increasing parameters exponentially. One area of inspiration for research is the acquisition of knowledge, which is addressed in the paper^40^ for learning small/intensive models for inference in recommender systems. The main idea is to train a smaller student model that absorbs knowledge from the larger teacher model.

Given that inference time is critical for real-time applications on a million/billion-user scale, this is another inspiring topic for future research. The next promising issue is to pay attention to compression methods^39^. High-dimensional input data can be compressed to reduce computational space and time during model learning.

## Data Availability

Datasets that have been used for experiments in this paper are available at: https://analyse.kmi.open.ac.uk/open_dataset.

## Abbreviations

LSTM: Long and short term memory
GRU: Gated recurrent unit
MLP: Multi-layer perceptron
DLS: Deep learning system
DSP: Discriminative sequential pattern
RNN: Recurrent recommender network
AROLS: Adaptive recommendation based on online learning style
CF: Collaborative filtering
ARM: Association rules mining
OLS: Online learning style
CNN: Convolutional neural network
RBM: Restricted boltzmann machine
OULAD: Open university learning analytics dataset

## References

[1] Dascalu M-I (2016). Educational recommender systems and their application in lifelong learning. Behav. Inform. Technol..

[2] Tarus JK, Niu Z, Yousif A (2017). A hybrid knowledge-based recommender system for e-learning based on ontology and sequential pattern mining. Futur. Gener. Comput. Syst..

[3] Zhang S (2019). Deep learning based recommender system: A survey and new perspectives. ACM Comput. Surv..

[4] Quadrana M, Cremonesi P, Jannach D (2018). Sequence-aware recommender systems. ACM Comput. Surv..

[5] Hidasi, B., *et al*., *Session-based recommendations with recurrent neural networks.* arXiv Preprint at http://arxiv.org/abs/quant-ph/1511.06939, (2015).

[6] Cheng, H.-T., et al. *Wide & Deep Learning for Recommender Systems*. In *Proc. 1st Workshop on Deep Learning for Recommender Systems*. (2016).

[7] Qiao C, Hu X, Qiao C, Hu X (2018). Discovering student behavior patterns from event logs: Preliminary results on a novel probabilistic latent variable model. 2018 Ieee 18th International Conference on Advanced Learning Technologies (icalt).

[8] Park SE, Lee S, Lee S-G, Park SE, Lee S, Lee S-G (2011). Session-based collaborative filtering for predicting the next song. 2011 First ACIS/JNU International Conference on Computers, Networks, Systems and Industrial Engineering.

[9] Moore JL (2013). Taste over time: The temporal dynamics of user preferences. ISMIR.

[10] Hu, L., et al. Diversifying Personalized Recommendation with User-session Context. In *International Joint Conference on Artificial Intelligence* 1858-1864 (Melbourne, Australia,2017).

[11] Ludewig M, Jannach D (2018). Evaluation of session-based recommendation algorithms. User Model. User-Adap. Inter..

[12] Rodríguez P (2017). An educational recommender system based on argumentation theory. AI Commun..

[13] IEEE Learning Technology Standards Committee. *IEEE Standard for Learning Object Metadata*. IEEE Standard 1484 **12** 1 (2002)

[14] Covington, P., J. Adams, and E. Sargin. *Deep neural networks for youtube recommendations*. In *Proc. 10th ACM Conference on Recommender Systems* (2016).

[15] Yago H (2018). On-smmile: Ontology network-based student model for multiple learning environments. Data Knowl. Eng..

[16] Serrà, J. and A. Karatzoglou. *Getting deep recommenders fit: Bloom embeddings for sparse binary input/output networks*. In *Proc. Eleventh ACM Conference on Recommender Systems*. (2017).

[17] Bourkoukou O, El Bachari E (2018). Toward a hybrid recommender system for e-learning personnalization based on data mining techniques. IJOIV: Int. J. Inform. Vis..

[18] Felder RM (2002). Learning and Teaching Styles In Engineering Education.

[19] Gulzar Z, Leema AA, Deepak G (2018). Pcrs: Personalized course recommender system based on hybrid approach. Procedia Comput. Sci..

[20] Hagemann N, O’Mahony MP, Smyth B, Hagemann N, O’Mahony MP, Smyth B (2018). Module advisor: Guiding students with recommendations. Intelligent Tutoring Systems: 14 International Conference, ITS 2018, Montreal, QC, Cnada 11-15, 2018, Proceedings 14. 2018.

[21] Tseng H-C, Tseng H-C (2017). Building an online adaptive learning and recommendation platform. Emerging Technologies for Education: First International Symposium, SETE 2016, Held in Conjunction with ICWL 2016, Rome, Italy, October 26–29, 2016, Revised Selected Papers 1.

[22] Alinani K (2016). Heterogeneous educational resource recommender system based on user preferences. Int. J. Auton. Adapt. Commun. Syst..

[23] Bourkoukou O, Achbarou O (2018). Weighting based approach for learning resources recommendations. JOIV Int. J. Inform. Vis..

[24] Thanh-Nhan H-L, Huy-Thap L, Thai-Nghe N, Thanh-Nhan H-L, Huy-Thap L, Thai-Nghe N (2017). Toward integrating social networks into intelligent tutoring systems. 2017 9th International Conference on Knowledge and Systems Engineering (KSE).

[25] Pupara K, Nuankaew W, Nuankaew P, Pupara K, Nuankaew W, Nuankaew P (2016). An institution recommender system based on student context and educational institution in a mobile environment. 2016 International Computer Science and Engineering Conference (ICSEC).

[26] Duque Méndez ND, Rodríguez Marín PA, Ovalle Carranza DA, Costa A, Julian V, Novais P (2018). Intelligent personal assistant for educational material recommendation based on CBR. Personal Assistants: Emerging Computational Technologies.

[27] Rossille D, Laurent J-F, Burgun A (2005). Modelling a decision-support system for oncology using rule-based and case-based reasoning methodologies. Int. J. Med. Inform..

[28] Neto J, Neto J (2018). Multi-agent web recommender system for online educational environments. Trends in Cyber-Physical Multi-Agent Systems. The PAAMS Collection-15th International Conference, PAAMS 2017 15.

[29] Rodríguez P, Duque N, Rodríguez S, Casillas J, Martínez-López FJ, Vicari R, De la Prieta F (2013). Integral multi-agent model recommendation of learning objects, for students and teachers. Management Intelligent Systems: Second International Symposium.

[30] Paradarami TK, Bastian ND, Wightman JL (2017). A hybrid recommender system using artificial neural networks. Expert Syst. Appl..

[31] Wang X, Wang X (2017). E-learning recommendation framework based on deep learning. 2017 IEEE International Conference on Systems, Man, and Cybernetics (SMC).

[32] Cho, K., et al., *Learning phrase representations using RNN encoder-decoder for statistical machine translation.* arXiv Preprint at http://arxiv.org/abs/quant-ph/1406.1078 (2014).

[33] Zhang H (2019). MOOCRC: A highly accurate resource recommendation model for use in MOOC environments. Mobile Netw. Appl..

[34] Najafabadi MM (2015). Deep learning applications and challenges in big data analytics. J. Big Data.

[35] Wang J, Wang J (2020). Attention-based CNN for personalized course recommendations for MOOC learners. 2020 International Symposium on Educational Technology (ISET).

[36] Ahmadian Yazdi H, Seyyed Mahdavi Chabok SJ, KheirAbadi M (2023). Effective data reduction for time-aware recommender systems. Control Opt. Appl. Math..

[37] Li R, Patnaik S, Jain V (2019). Online learning style modeling for course recommendation. Recent Developments in Intelligent Computing, Communication and Devices: Proceedings of ICCD 2017.

[38] Hagemann, N., M.P. O'Mahony, and B. Smyth. *Visualising module dependencies in academic recommendations*. In *Proc. 24th International Conference on Intelligent User Interfaces: Companion*. (2019).

[39] Tseng H-C, Ting-Ting W, Gennari R, Huang Y-M, Xie H, Cao Y (2016). Building an online adaptive learning and recommendation platform. International Symposium on Emerging Technologies for Education.

[40] Kuznetsov S, Kuznetsov S (2016). Reducing cold start problems in educational recommender systems. 2016 International Joint Conference on Neural Networks (IJCNN).

[41] Felder RM, Silverman LK (1988). Learning and teaching styles in engineering education. Eng. Educ..

[42] Zhang H (2019). MOOCRC: A highly accurate resource recommendation model for use in MOOC environments. Mobile Netw. Appl..

[43] Bonyani M (2023). DIPNet: Driver intention prediction for a safe takeover transition in autonomous vehicles. IET Intell. Trans. Syst..

[44] Bonyani M, Ghanbari M, Rad A (2022). Different gaze direction (DGNet) collaborative learning for iris segmentation. SSRN Electron. J..

[45] Allen DM (1974). The relationship between variable selection and data agumentation and a method for prediction. Technometrics.

[46] Kuzilek J, Hlosta M, Zdrahal Z (2017). Open university learning analytics dataset. Sci. Data.

[47] Winata GI, Kampman OP, Fung P, Winata GI, Kampman OP, Fung P (2018). Attention-based lstm for psychological stress detection from spoken language using distant supervision. 2018 IEEE International Conference on Acoustics, Speech and Signal Processing (ICASSP).

[48] Luong HH, Luong HH (2022). Feature selection using correlation matrix on metagenomic data with pearson enhancing inflammatory bowel disease prediction. International Conference on Artificial Intelligence for Smart Community: AISC 2020, 17–18 December, Universiti Teknologi Petronas, Malaysia.

[49] Bonyani M, Jahangard S, Daneshmand M (2021). Persian handwritten digit, character and word recognition using deep learning. Int. J. Doc. Anal. Recognit..

[50] Wu, C.-Y., et al. *Recurrent recommender networks*. In *Proc. Tenth ACM International Conference on web Search and Data Mining*. (2017).

[51] Hill W, Hill W (1995). Recommending and evaluating choices in a virtual community of use. Proceedings of the SIGCHI Conference on Human Factors in Computing Systems.

[52] Yan L, Yan L (2021). Learning Resource Recommendation in E-Learning Systems Based on Online Learning Style. Knowledge Science, Engineering and Management: 14th International Conference, KSEM 2021, Tokyo, Japan, August 14–16, 2021, Proceedings, Part III.

[53] Ahmadian Yazdi H, Seyyed Mahdavi Chabok SJ, Kheirabadi M (2022). Dynamic educational recommender system based on improved recurrent neural networks using attention technique. Appl. Artif. Intell..

